# The evolution of antibiotic susceptibility and resistance during the formation of *Escherichia coli* biofilms in the absence of antibiotics

**DOI:** 10.1186/1471-2148-13-22

**Published:** 2013-01-28

**Authors:** Jabus G Tyerman, José M Ponciano, Paul Joyce, Larry J Forney, Luke J Harmon

**Affiliations:** 1 Department of Biological Sciences, University of Idaho, Campus Box 3051, Moscow, ID, 83843, USA; 2 Initiative for Bioinformatics and Evolutionary Studies (IBEST), University of Idaho, Moscow, ID, 83844, USA; 3 Department of Biology, University of Florida, Gainesville, FL, USA; 4 Departments of Mathematics, University of Idaho, Moscow, Moscow, ID, 83844, USA; 5 Departments of Statistics, University of Idaho, Moscow, ID, 83844, USA; 6 Current address: Genomatica, Inc., 10520 Wateridge Circle, San Diego, CA, 92121, USA

**Keywords:** Evolution, Antibiotic resistance, Bacterial biofilms, Mutations, Diversity

## Abstract

**Background:**

Explanations for bacterial biofilm persistence during antibiotic treatment typically depend on non-genetic mechanisms, and rarely consider the contribution of evolutionary processes.

**Results:**

Using *Escherichia coli* biofilms, we demonstrate that heritable variation for broad-spectrum antibiotic resistance can arise and accumulate rapidly during biofilm development, even in the absence of antibiotic selection.

**Conclusions:**

Our results demonstrate the rapid *de novo* evolution of heritable variation in antibiotic sensitivity and resistance during *E. coli* biofilm development. We suggest that evolutionary processes, whether genetic drift or natural selection, should be considered as a factor to explain the elevated tolerance to antibiotics typically observed in bacterial biofilms. This could be an under-appreciated mechanism that accounts why biofilm populations are, in general, highly resistant to antibiotic treatment.

## Background

Bacteria that form biofilms have been shown to be highly resistant to antimicrobial therapy [1-3] and contribute to the chronic nature of many bacterial infections in humans because cells in biofilms are highly resistant to antibiotic treatment (e.g. [4,5]). Developing effective treatments for biofilm-related infections requires an understanding of the processes that lead biofilms to persist in the face of antimicrobial treatment [6].

There are multiple hypotheses to explain biofilm persistence during antibiotic treatment [7-10]. Physical factors, like diffusion limitation, may prevent antibiotic concentrations from reaching inhibitory or lethal levels within biofilms [11,12]. However, several studies report biofilm persistence despite substantial antibiotic diffusion (e.g. [13,14], reviewed in [1,15]). Another hypothesis posits that non-genetic phenotypic heterogeneity, including the plastic expression of phenotypes that are resistant to antibiotics, may occur in biofilms. For example, resource gradients may lead cells to experience different microenvironments and thus express distinct phenotypes in different parts of a biofilm [1,9]. In particular, a “persister” phenotype has been hypothesized to arise in biofilms and provide immunity against antibiotics [16]. These persister cells are postulated to have the ability to reconstitute biofilms upon release from antibiotic threat [16-19].

Alternatively, bacteria cultured as biofilms may evolve heritable variation for resistance to antibiotics *de novo*[7]. We know that evolutionary change can occur rapidly within tens to hundreds of generations [20], which are time scales relevant to medical treatment of infectious diseases [21]. Furthermore, bacteria in biofilms have huge population sizes so that many new mutations will arise over relatively short time scales. It is possible that antibiotic resistance might arise in bacterial biofilms through straightforward population genetic processes. We suggest that, given enough time, variation in antibiotic resistance may arise in biofilms even in the absence of antibiotic selection. This could happen due to the accumulation of neutral variation or as a result of selection for phenotypes that by happenstance are correlated with antibiotic resistance (e.g. [22]).

Evolutionary change in biofilms is plausible since recent studies have shown tremendous phenotypic variation among cells isolated from biofilms growing in patients [23-25] and demonstrated heritable variation in traits within experimentally cultured biofilm populations [26-32]. However, few hypotheses about antibiotic resistance in biofilms invoke evolutionary change as an alternative explanation for biofilm persistence during antibiotic treatment (but see [7,8,27]). Evolution could involve mutations that convey resistance to single antibiotics (specialized resistance) or to whole suites of antibiotics (broad-spectrum resistance [8]). The occurrence of variants resistant to antibiotics may provide an “insurance effect” [30,32,33] by creating subpopulations of cells that can survive or even proliferate should the biofilm come under antibiotic assault. Resistant variants could thus facilitate reconstitution of bacterial biofilm populations following cessation of antibiotic treatment.

Documenting whether genetic variation for antibiotic resistance arises during the course of biofilm development is an important first step to exploring biofilm persistence in the face of antibiotic treatment. As we suggest, evolutionary explanations predict that genetic variation in the susceptibility to antibiotics will arise in biofilms, and that the frequency of antibiotic resistant cells will increase through time. Here we demonstrate the rapid evolution of heritable variation for broad-spectrum antibiotic resistance during the course of biofilm development by *E. coli*. We tested three hypotheses in this work: (1) heritable variation for antibiotic resistance evolves during biofilm development; (2) this variation includes both resistant and susceptible mutants to a range of antibiotics; and (3) phenotypic variation in biofilms increases through time. We found evidence for both (1) and (2) but not (3). Variation in antibiotic resistance emerged within 15 days of biofilm growth, a time frame that is consistent with many common bacterial infections. Thus, these findings have important implications for the development of treatments for bacteria that form biofilms during infection.

## Results and discussion

We cultured *Escherichia coli* K12 MG1655 as biofilms in the absence of antibiotics. Briefly, we inoculated *E. coli* into flow-cells and cultured biofilms using minimal medium with glucose as a sole carbon source. We sampled biofilm populations at 15, 30 and 60 days and isolated ten bacterial clones from three replicate flow cells at each time point. Using the Kirby-Bauer disc diffusion method [34], we characterized each bacterial clone for resistance to twelve antibiotics by measuring the diameters of zones of (growth) inhibition (ZOI) for each clone on each antibiotic (Table 1; see detailed methods below).

**Table 1:** Summary of the antibiotics used in this study

**Antibiotic class**	**Antibiotic**	**Abbreviation**^**1**^
Aminoglycoside	Streptomycin	S10
	Gentamicin	GM10
	Kanamycin	K30
Quinolone	Naladixic acid	NA30
	Ciprozone	CIP5
Beta-lactam	Ampicillin with Sulbactam	SAM20
	Cefoperazone	CFP75
Semi-synthetic (rifamycin group)	Rifampin	RA5
Cationic basic protein	Polymyxin B	PB300
Macrolide	Erythromycin	E15
Glycopeptide	Vancomycin	VA30
Polyketide	Tetracycline	TE30

^1^Based on Sensi-disks from BD. Numbers following letters indicate ug of antibiotic applied to each disk.

**Figure 1:**
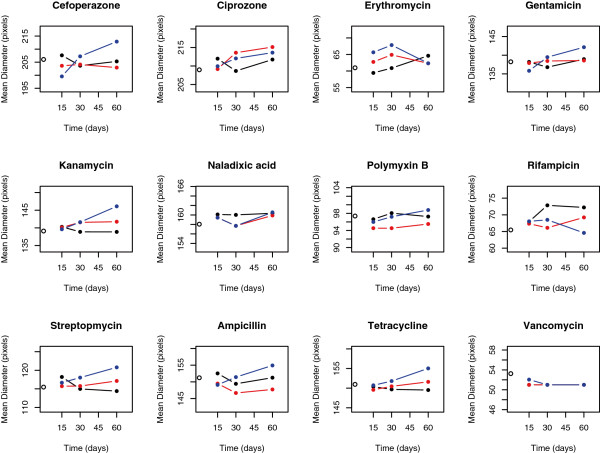
**Mean diameter (in pixels) of the zone of inhibition (ZOI), a measure of antibiotic resistance, across ancestor (time zero) and bacteria isolated from biofilms at 15, 30, and 60 days.** Individual replicates appear as distinct colors connected with a line. The ZOI of the ancestor is plotted at time 0 as an open circle.

**Figure 2:**
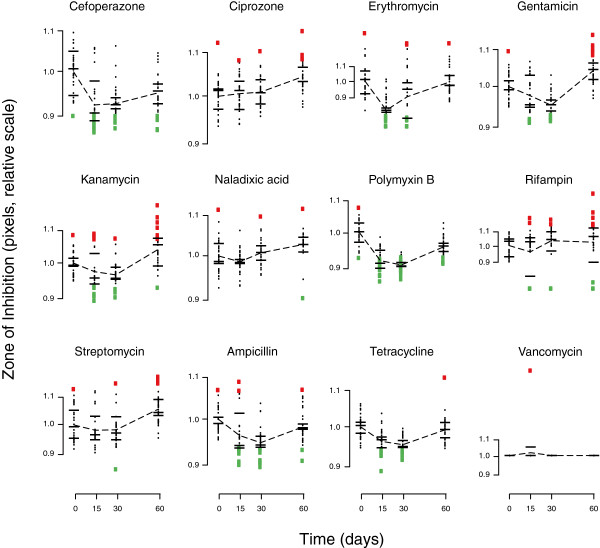
**Raw data for zone of inhibition, a measure of antibiotic resistance, across ancestor (time zero) and bacteria isolated from biofilms at 15, 30, and 60 days.** Individual clones appear as dots. Red or green squares denote sensitive and resistant forms, respectively, determined as datapoints that are more than two standard deviations above or below the mean. Means for each replicate are marked with black bars and overall means connected with a dotted line. Antibiotics in the same class are followed by matching symbols.

**Figure 3:**
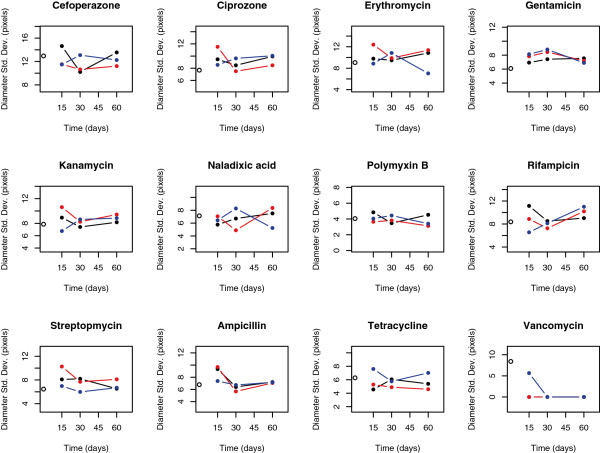
**Standard deviation (in pixels) of the diameters of zones of inhibition (ZOI) across ancestor (time zero) and bacteria isolated from biofilms at 15, 30, and 60 days.** Individual replicates appear as distinct colors connected with a line. The variance across ZOI measurements of the ancestor is plotted at time 0 as an open circle.

We tested whether heritable variation for antibiotic resistance evolved during biofilm development by assessing change in mean ZOI for the biofilm-derived clones relative to the ancestor. We observed the evolution of statistically significant differences in antibiotic resistance in biofilms (one-way MANOVA across all levels of biofilm replicate × time, Wilks’ = 0.016, P < 0.0001; Figure 1), with clones that were more sensitive or more resistant appearing independently in each of our replicates (Figure 2; see Additional file 1: Figure S1 for evidence that this variation is heritable, and Additional file 1: Table S1 for an analysis of correlations across different antibiotics). The data used for the analysis was formatted to create a balanced design matrix. Using a set of nine planned contrasts, we found significant changes in the mean evolved resistance for several antibiotics through time (Table 2). Many evolved clones also showed increased susceptibility to antibiotics. Although resistant clones were uncommon in our experiments, when they appeared in evolved biofilms, they did so at notable frequencies (Figure 2). For some combinations of antibiotics and sampling times, multiple samples from evolved biofilms showed higher variability than seen among multiple samples from the ancestral clone (Figure 3). These findings support hypotheses 1 and 2; genetic variation in levels of resistance to antibiotics evolves during biofilm development in the absence of antibiotics, and this variation includes both resistant and sensitive clones. There was no evidence of increased variation through time in our data (hypothesis 3; linear regression of total multivariate phenotypic variation [disparity] among clones vs. biofilm age, P > 0.05; number of resistant or sensitive clones vs. biofilm age, P > 0.05).

**Table 2:** Results of planned contrasts following one-way MANOVA on mean ZOI across biofilm replicates and time

**Contrast**	**Antibiotics that differ significantly**
Ancestor vs. 15 days	CFP75↓, PB300↓
Ancestor vs. 30 days	CFP75↓, GM10↓, PB300↓, SAM20↓, TE30↓
Ancestor vs. 60 days	none
15 days vs. 30 days	GM10↑
15 days vs. 60 days	CIP5↓, E15↓, GM10↓, K30↓, NA30↓, PB300↓, S10↓, TE30↓
30 days vs. 60 days	CIP5↓, GM10↓, K30↓, PB300↓, S10↓, SAM20↓, TE30↓
Biofilm 1 vs. biofilm 2	none
Biofilm 1 vs. biofilm 3	PB300↑
Biofilm 2 vs. biofilm 3	none

Symbols denote direction of change: ↓ = smaller ZOI for latter group, ↑ = larger ZOI for latter group.

Our results suggest that antibiotic resistance and susceptibility can rapidly evolve in biofilms over relatively short time scales (<15 days), which begs the question of how these rates of mutation accumulation compare to those in well-mixed liquid cultures that support exponential growth. Such a comparison is difficult because estimating the “mutation rate” in the spatially structured bacterial cells of biofilms is problematic. Mutation rates are almost always calculated and compared on a “per-generation” basis (e.g. [35,36]), but rates of bacterial cell division in biofilms vary widely depending on location within the biofilm matrix. This variation in cellular growth rates is a consequence of nutrient depletion and the creation of strong gradients of substrates, electron acceptors and other resources within the spatially structured environment of biofilms [37,38]. These gradients cause growth rates to vary tremendously within biofilms, such that cells deep within the biofilm matrix may not divide at all [39]. Because of this, mutation frequency cannot be expressed in the same terms, i.e., per generation, as in well-mixed liquid cultures; nor can one calculate a meaningful population-wide average growth rate for cells in biofilms. One can imagine applying models that account for differential growth in biofilms (e.g., [40]), and then using current data to calculate mutation rates that can be compared to rates in well-mixed cultures. However, such calculations require data about mutation rates in in non-growing bacterial cells that is largely lacking, so direct and simple comparisons between biofilms and well-mixed cultures are not possible at this time.

The evolution of antibiotic resistance and susceptibility in bacterial biofilms involves the interaction between mutation, selection, genetic drift, and spatial structure [26,40]. The data presented here cannot determine the importance of these multiple explanatory factors. It seems likely that evolution in biofilms typically occurs under conditions contrary to what is typically assumed in standard population genetics theory (e.g. strong selection and weak mutation) and rather involves strong mutational mechanisms typical in bacteria under stress [41] coupled with weak selection (see also [26]). Future work combining spatially explicit models for biofilm growth (e.g. [40,42]) with model-based estimates of mutation rates and effect sizes for bacteria (e.g. [43]) would provide more insight into the details of evolution in biofilms.

## Conclusions

These data show the rapid *de novo* evolution of heritable variation in antibiotic sensitivity and resistance during *E. coli* biofilm development. We suggest that evolutionary processes, whether genetic drift or natural selection, should be considered as a factor to explain the elevated tolerance to antibiotics typically observed in bacterial biofilms. We do not yet know whether evolution of antibiotic resistance requires high rates of mutation as can arise in biofilms (e.g. [44]) or can be explained by normal mutation rates in bacteria. In either case, biofilms quickly evolve high levels of variation in antibiotic resistance. We hypothesize that rare, highly resistant variants may allow biofilms to regrow following antibiotic treatment. This mechanism is an important potential explanation for why biofilm populations are, in general, highly resistant to antibiotic treatment.

## Methods

### Strain, media and growth conditions

Bacterial biofilms were grown as described by Ponciano *et al.*[26]. *Escherichia coli* K12 MG1655 was grown in minimal salts media (M9) augmented with vitamins and trace elements with 0.05% glucose as the carbon source. The inoculum for flow cells was prepared by inoculating 10 ml of minimal medium with a scraping from a -80°C freezer stock and incubating the culture for 24 h at 37°C. Biofilms were cultured in flow-cells that had been sterilized by flowing 5% bleach for >24 h, followed by rinsing with minimal medium for 24 h. A 100 ul inoculum was introduced to each flow cell using a sterile syringe and needle. Bacterial cells were allowed to settle for 6 h before the flow was restarted (with a mean hydraulic retention time of 2.5 h). The biofilms were cultured for 15, 30 or 60 days prior to sampling them through a port on the upper surface of the flow-cell using a syringe and needle. Each sample was vortexed for 1 minute, then serially diluted in saline and plated on minimal medium solidified with agar. Ten randomly chosen clones were obtained from each of three replicate biofilms sampled at four times: 0, 15, 30 or 60 days. The 0d samples are referred to as “ancestors”. All clones were grown overnight in minimal medium and archived as glycerol stock cultures at -80°C.

### Antibiotic sensitivity

We determined the sensitivity of ancestral and biofilm clones (15, 30 and 60 days old) to 12 antibiotics using the Kirby-Bauer disk method. The antibiotics (Table 1) were selected to target a range of cellular processes. Individual clones were grown in minimal medium for 24 h (final optical density at 600 nm = 0.15-0.2) and spread on Mueller-Hinton agar using sterile cotton swabs to form a lawn. After allowing the plate to dry for about 10 minutes, antibiotic-infused disks (Sensi-Disks, BD, New Jersey) were placed on the plates, which were then incubated for 18 h at 37°C. We photographed plates from a standard distance and measured the zones of (growth) inhibition (ZOI) for each antibiotic disk using ImageJ software available for download from NIH (http://rsbweb.nih.gov/ij/).

Antibiotic resistance was quantified as the diameter (in pixels) of the ZOI around the antibiotic-infused disks. Susceptible clones had relatively larger ZOI, while resistant clones had relatively smaller ZOI. For each bacterial clone, we replicated the resistance score for each antibiotic three times by growing three independent cultures from the frozen stock of that clone. For each independent replicate, we used the mean ZOI from two antibiotic disks. For each antibiotic disk, we scored the ZOI as the mean of three arbitrarily drawn diameters across the ZOI. Thus, each resistance score represents a mean of 3 × 2 × 3 = 18 individual measurements. Finally, at each time point we measured two replicates of the ancestor as a control. At each sampling time we standardized scores by dividing each by the mean score of the control to reduce variation introduced by day-to-day fluctuations in media (i.e., agar thickness, dryness, concentration, etc.).

### Analysis

To test the hypothesis that heritable variation for antibiotic resistance arose during biofilm development, we carried out a one-way MANOVA across all biofilms and time points simultaneously (Table 1). This MANOVA used ZOI diameter across all antibiotics as a response variable, and a concatenated variable of biofilm identity and time as predictor variable (the 10 levels of the predictor variable were then: ancestral line at 0 days, Biofilm replicate 1 at 15, 30, and 60 days, Biofilm replicate 2 at 15, 30, and 60 days, and biofilm replicate 3 at 15, 30, and 60 days). We also subjected these data to a set of nine planned contrasts (all paired comparisons of the ancestral population and all biofilms from 15, 30, and 60 days, as well as all paired comparisons between the three biofilm replicates; see Table 2). To account for an inflated Type I error associated with multiple comparisons, we computed the conservative simultaneous confidence intervals for each contrast [45].

We identified sensitive and resistant forms, respectively, as clones whose mean ZOI was more than two standard deviations above or below the mean of the ancestor. To test for increasing variation through time, we used linear regression to compare both total multivariate phenotypic variation [disparity] among clones and the number of resistant or sensitive clones to biofilm age.

All analyses were conducted using R (version 2.12.2 [46]).

## Competing interests

The authors declare no competing interests.

## Authors’ contributions

JGT, PJ, LJF, and LJH designed the study; JGT collected the data; JGT and JMP analyzed and interpreted results; and JGT, JMP, LJF, and LJH wrote the paper. All authors read and approved the final manuscript.

## Supplementary Material

Additional file 1: Figure S1. Analysis of the heritability of the resistance phenotype across clones. If the phenotype is stably inherited, then it would be expected that across all treatments, the two cultures would show the same resistance phenotype (i.e. a difference in mean ZOI diameter of 0) despite experiencing slightly different growth. The figure below depicts the histogram of the difference in the mean resistance phenotype (diameter of ZOI) between two independent overnight cultures for each clone, across all treatments. The mean difference was not significantly different from 0 [mean = -0.035, P = 0.52, n = 3147, confidence interval for the mean = (-1.07, 0.94)], which strongly suggests that the diversity in resistance phenotypes is due to heritable changes. **Table S1.** Spearman rank correlations of antibiotic resistance across different antibiotics (see Additional file 1: Table S1 for abbreviations). Correlations were calculated across all individual clones pooled across time intervals (n = 90; 3 replicates x 10 clones / replicate x 3 time points). Significant (p < 0.05) correlations are noted, with * P < 0.05, ** P < 0.01, *** P < 0.001. Click here for file
