# Supplementary material for: The evolution of antibiotic susceptibility and resistance during the formation of Escherichia coli biofilms in the absence of antibiotics
Source: BMC Evol Biol. 2013 Jan 28;13:22. doi: 10.1186/1471-2148-13-22 (PMC3568021; doi:10.1186/1471-2148-13-22)
Supplement: Additional file 1: Figure S1. — Analysis of the heritability of the resistance phenotype across clones. If the phenotype is stably inherited, then it would be expected that across all treatments, the two cultures would show the same resistance phenotype (i.e. a difference in mean ZOI diameter of 0) despite experiencing slightly different growth. The figure below depicts the histogram of the difference in the mean resistance phenotype (diameter of ZOI) between two independent overnight cultures for each clone, across all treatments. The mean difference was not significantly different from 0 [mean = -0.035, P = 0.52, n = 3147, confidence interval for the mean = (-1.07, 0.94)], which strongly suggests that the diversity in resistance phenotypes is due to heritable changes. Table S1. Spearman rank correlations of antibiotic resistance across different antibiotics (see Additional file 1: Table S1 for abbreviations). Correlations were calculated across all individual clones pooled across time intervals (n = 90; 3 replicates x 10 clones / replicate x 3 time points). Significant (p < 0.05) correlations are noted, with * P < 0.05, ** P < 0.01, *** P < 0.001. [file 1471-2148-13-22-S1.doc]

Appendix 1:

Figure S1. Analysis of the heritability of the resistance phenotype across clones. If the phenotype is stably inherited, then it would be expected that across all treatments, the two cultures would show the same resistance phenotype (i.e. a difference in mean ZOI diameter of 0) despite experiencing slightly different growth. The figure below depicts the histogram of the difference in the mean resistance phenotype (diameter of ZOI) between two independent overnight cultures for each clone, across all treatments. The mean difference was not significantly different from 0 [mean = -0.035, P = 0.52, n=3147, confidence interval for the mean = (-1.07, 0.94)], which strongly suggests that the diversity in resistance phenotypes is due to heritable changes.

Table S1. Spearman rank correlations of antibiotic resistance across different antibiotics (see Table S1 for abbreviations). Correlations were calculated across all individual clones pooled across time intervals (n = 90; 3 replicates x 10 clones / replicate x 3 time points). Significant (p<0.05) correlations are noted, with * P < 0.05, ** P < 0.01, *** P < 0.001.

|  | cfp75 | cip5 | e15 | gm10 | k30 | na30 | pb300 | ra5 | s10 | sam20 | te30 | va30 |
| --- | --- | --- | --- | --- | --- | --- | --- | --- | --- | --- | --- | --- |
| cfp75 | - |  |  |  |  |  |  |  |  |  |  |  |
| cip5 | 0.70* | - |  |  |  |  |  |  |  |  |  |  |
| e15 | -0.38 | -0.34 | - |  |  |  |  |  |  |  |  |  |
| gm10 | 0.79** | 0.86*** | -0.22 | - |  |  |  |  |  |  |  |  |
| k30 | 0.89*** | 0.77** | -0.56 | 0.81** | - |  |  |  |  |  |  |  |
| na30 | 0.39 | 0.43 | -0.62* | 0.33 | 0.56 | - |  |  |  |  |  |  |
| pb300 | 0.75** | 0.75** | -0.34 | 0.58 | 0.73** | 0.29 | - |  |  |  |  |  |
| ra5 | -0.73** | -0.67* | 0.06 | -0.73** | -0.78** | -0.35 | -0.69* | - |  |  |  |  |
| s10 | 0.84** | 0.81** | -0.21 | 0.87*** | 0.71* | 0.32 | 0.67* | -0.69* | - |  |  |  |
| sam20 | 0.87*** | 0.82** | -0.12 | 0.81** | 0.73** | 0.25 | 0.74** | -0.69* | 0.84** | - |  |  |
| te30 | 0.75** | 0.76** | -0.59* | 0.73** | 0.82** | 0.72* | 0.54 | -0.62* | 0.74** | 0.62* | - |  |
| va30 | -0.13 | -0.31 | 0.39 | -0.22 | -0.04 | -0.22 | -0.04 | -0.24 | -0.31 | -0.13 | -0.48 | - |
